# Outcomes of endometrial cancer prevention strategies in patients with Lynch syndrome: a nationwide cohort study in the Netherlands

**DOI:** 10.1016/j.eclinm.2024.103006

**Published:** 2024-12-21

**Authors:** Ellis L. Eikenboom, Lotte van Leeuwen, Floris Groenendijk, Jorien M. Woolderink, Anne M. Van Altena, Monique E. Van Leerdam, Manon C.W. Spaander, Helena C. van Doorn, Anja Wagner, collaborative investigators from the Dutch Foundation for Detection of Hereditary Tumors, M.C. Breijer, A.S. Tjalsma, F. Vork, H.P.M. Smedts, J. van der Velden, M.M.A. Brood-van Zanten, J.E. van de Riet, A.L.M. Oei, H. Kessel, P.M.L.H. Vencken, M.P.L.M. Snijders, R.H.M. Hermans, A. Bouman, H.W. Ünsalan, A.M.G. van de Swaluw, G.M. Plaisier, H.C. van Doorn, K. van den Berg, W. Hofhuis, Y.A.J.M. Dabekausen, P.R. Kolk, H.T.C. Nagel, A.M.L.D. van Haaften-de Jong, A.C. van Hof, M. van den Hende, J. Kaijser, H.H. de Haan, R.A. Smit, M.W.G. Moonen-Delarue, J.J. Beltman, J.E. Martens, R. Kruitwagen, J.M. van der Ploeg, J.M. Woolderink, S.F.P.J. Coppus, M.J. Duk, M.J.A. Apperloo, C.M. Koopmans, C.C.M. Buis, H. van Meurs, E.C. Dul, B.B.J. Hermsen, A.M. van Altena, A. Baalbergen, A.A. van Ginkel-Terng, M. Baas, P. van Greunen, C.M.W.H. Smeets, H. Knipscheer, J.E. Martens, C. Schmeink, M.D. van der Laan, E.J.M. van Es, J.E.W. van Dijk, F.M.F. Rosier-van Dunné, H. Nijman, C.G. Gerestein, D. Boskamp, E.C.A.H. Scheers, M. Verbruggen, L.R. Bartelink, C.B.M. Kruijdenberg, J.M. Briët, B. Visschers, M. Engelen

**Affiliations:** a Department of Clinical Genetics, Erasmus MC Cancer Institute, Rotterdam, the Netherlands; b Department of Gastroenterology & Hepatology, Erasmus MC Cancer Institute, Rotterdam, the Netherlands; c Department of Pathology, Erasmus MC Cancer Institute, Rotterdam, the Netherlands; d Department of Obstetrics and Gynecology, Martini Hospital Groningen, Groningen, the Netherlands; e Division of Gynecologic Oncology, Department of Obstetrics and Gynecology, Radboud University Medical Centre, Nijmegen, the Netherlands; f Department of Gastroenterology and Hepatology, Leiden University Medical Center, the Netherlands; g Department of Gastrointestinal Oncology, Netherlands Cancer Institute Amsterdam, the Netherlands; h Department of Gynecologic Oncology, Erasmus MC Cancer Institute, Rotterdam, the Netherlands

**Keywords:** Lynch syndrome, Gynaecological surveillance, Endometrial carcinoma

## Abstract

**Background:**

Female Lynch syndrome carriers have an increased risk of developing endometrial cancer. Regardless, research on endometrial carcinoma tumorigenesis is scarce and no uniform, evidence-based gynaecological management guidelines exist. We therefore described gynaecological surveillance and surgery outcomes in a nation-wide Lynch syndrome cohort.

**Methods:**

For this retrospective cohort study, female Lynch syndrome carriers, prospectively registered in the Dutch Lynch syndrome database (StOET), were included up to February 28th 2022. Carriers were linked to the Dutch national pathology (PALGA) database. The number of carriers with/without gynaecological surveillance, number of index carriers with endometrial carcinoma before Lynch syndrome diagnosis were assessed, as well as uptake of risk-reducing surgery and characteristics of endometrial carcinomas including the requisite for adjuvant therapy according to current guidelines. Overall survival after endometrial carcinoma diagnosis was analyzed using Kaplan Meier time to event analyses, cumulative incidence was calculated after adjusting for competing risks (death and prophylactic hysterectomy).

**Findings:**

In total, 1046 registered female Lynch syndrome carriers were eligible for surveillance, of whom 313 (30.0%) did not have surveillance and 21.4% (n = 224 of 1046) opted for prophylactic hysterectomy. In carriers with surveillance, more cases of endometrial carcinoma and hyperplasia were found than in those without (37 endometrial carcinomas (7.3%) and 28 hyperplasias (5.5%) in 506 carriers with surveillance versus 14 (2.6%) and 4 (0.7%) in 540 carriers without surveillance, respectively); carriers with surveillance were generally younger than those without (median 56 years [IQR 48–65] versus median 65 years [IQR 49–75] at database assembly, respectively; p < 0.0001). Endometrial carcinomas were predominantly of endometrioid type and FIGO stage IA, regardless of surveillance. Adjuvant external beam radiotherapy was required in one patient in both groups. Overall survival after endometrial carcinoma diagnosis did not differ between carriers with or without surveillance or carriers with endometrial carcinoma before LS diagnosis (p = 0.51). For all endometrial carcinomas together, including index carriers, cumulative incidence was 22.7% at age 70.

**Interpretation:**

In a nation-wide cohort of Lynch syndrome carriers, nearly one-third of eligible carriers did not undergo gynaecological surveillance. Endometrial carcinomas diagnosed during surveillance were slightly more often stage FIGO IA, but this did not seem to substantially decrease the requisite for adjuvant therapy or affect overall survival, questioning effectiveness of current gynaecological management. Prospective research should further assess this, as well as patient preferences.

**Funding:**

None.

**Research in context**

**Evidence before this study**

Before analyzing the data of our nationwide cohort, we systematically searched PubMed on studies in English published until June 15 2021, where gynaecological surveillance in Lynch syndrome carriers was reported. The search was repeatedly updated, with the last search carried out on August 25 2024. As female Lynch syndrome carriers have an increased risk to develop gynaecological tumors (endometrial and ovarian carcinoma), all Dutch female Lynch syndrome carriers aged between 40 and 60 years of age are currently advised to visit the gynaecologist annually for a transvaginal ultrasound, endometrial biopsy, and assessment of the ovaries. Similarly, risk-reducing surgery (hysterectomy with or without bilateral salpingo-oophorectomy) can be considered to further decrease the risk of endometrial (and ovarian) carcinoma. However, gynaecological management strategies in Lynch syndrome carriers differ between countries, and limited evidence is available to support these guidelines.

**Added value of this study**

This nationwide, prospectively maintained cohort study, showed that nearly one-third of eligible women did not have gynaecological surveillance and that in total, slightly more than one out of five female Lynch syndrome carriers eventually chose for prophylactic surgery. Despite the fact that in carriers with surveillance, more endometrial carcinomas and hyperplasia were found, and endometrial carcinomas were found in a slightly earlier stage, this did not result in significantly less adjuvant therapy or a significantly increased overall survival. Previous research only showed low-quality and limited evidence that gynaecological surveillance could detect cancers in female Lynch syndrome carriers. A modeling study showed that surveillance could be cost-effective, but in practice, a significant survival benefit has not been shown so far. Our study questions the effectiveness of the current surveillance strategy.

**Implications of all the available evidence**

To our knowledge, this is the first nationwide study assessing gynaecological surveillance and prophylactic surgery outcomes in female Lynch syndrome carriers. The results of this study contribute to the debate questioning the effectiveness of current gynaecological management in female Lynch syndrome carriers. As endometrial biopsies are considered painful and possibly form a reason for women to opt out of surveillance, alternatives for gynaecological surveillance in this group of patients should be explored, as well as patient preferences on this matter. Similarly, future prospective studies in a larger group of female Lynch syndrome carriers are needed to conclusively determine the added value of current gynaecological surveillance and to explore reasons why women have declined surveillance earlier.

## Introduction

Lynch syndrome is one of the most prevalent hereditary cancer predisposition syndromes.^1^^,^^2^ Lynch syndrome is caused by pathogenic germline variants (PV) in DNA mismatch repair (MMR) genes *MLH1*, *MSH2*, *MSH6*, *PMS2* or by a deletion of the 3’ end of *EpCAM* (*TACSTD1*).^3^, ^4^, ^5^, ^6^, ^7^ Besides an increased risk of up to 57.1% to develop colorectal cancer (CRC; cumulative incidence at age 75 for *MLH1* up to 57.1%; *MSH2* up to 51.4%; *MSH6* up to 20.3%*; PMS2* up to 10.4%), female carriers are also at high risk to develop gynaecological malignancies, such as endometrial cancer and cancer in the ovaries (OC).^8^ The risks for endometrial carcinoma development by age 75 were found to be up to 37.0%, 48.9%, 41.1%, and 12.8% for *MLH1*, *MSH2*, *MSH6* and *PMS2* carriers, respectively. The risk of developing OC by age 75 was found to be 3.0–17.4% depending on the MMR gene involved (lowest percentage *PMS2*, highest *MSH2*).^8^

Development of surveillance strategies, such as biennial colonoscopy, led to a decrease in CRC-related deaths due to earlier discovery of CRC or its precursor lesions.^9^, ^10^, ^11^ However, global guidelines for gynaecological surveillance are diverse, due to a lack of insight into gynaecological carcinogenesis in Lynch syndrome carriers and the efficiency of gynaecological surveillance.^12^, ^13^, ^14^, ^15^, ^16^ A 2021 review by Ryan et al.^17^ comparing different gynaecological preventative strategies found that gynaecological surveillance detects cancers in female Lynch syndrome carriers, although evidence for this claim was limited and of low quality. It was not shown that gynaecological surveillance improved survival, or which form of gynaecological surveillance was best for this group of women.

Nevertheless, in the Netherlands, female Lynch syndrome carriers between 40 and 60 years are advised a yearly gynaecological examination with transvaginal ultrasound and endometrial biopsy, and optionally, assessment of the ovaries.^18^ Additionally, risk-reducing surgery such as a total hysterectomy and bilateral salpingo-oophorectomy can be considered after completion of childbearing, to further decrease the risk of gynaecological cancer development.^19^ However, this strategy is not extensively evaluated yet, due to a paucity of literature on this topic.^18^^,^^20^, ^21^, ^22^ Previously, our group assessed the performance of gynaecological Lynch syndrome surveillance in the South-West of The Netherlands and found that the examinations performed during these surveillance visits varied enormously, as well as time between subsequent surveillance visits.^23^

Additionally, we found that, despite enormous variations in performed tests and time-intervals, all endometrial carcinomas found during follow-up were diagnosed in International Federation of Gynecology and Obstetrics (FIGO) stage I. However, it is important to recognize that current guidelines recommend additional therapy in intermediate and high-risk low stage endometrial carcinomas, based on myometrial invasion, age, lymph vascular space invasion (LVSI), grade of differentiation and histological type.^24^^,^^25^ This also raised the question to what extent current gynaecological surveillance reduces the need for adjuvant treatment, so that carriers not only have better survival, but also less morbidity induced by adjuvant therapy.

Therefore, we performed a retrospective, nationwide study, with prospectively-managed data obtained in Dutch female Lynch syndrome carriers to assess these carriers’ uptake with regards to gynaecological surveillance and preventative surgery, as well as to describe the outcomes (hyperplasia and endometrial carcinoma) of the Dutch gynaecological management in this population. The percentage and stage of endometrial carcinomas identified and overall survival were compared between patients with and without surveillance. In patients with endometrioid endometrial carcinoma FIGO IA/IB, the percentage of carriers requiring adjuvant therapy (according to the latest guidelines) was compared between those who did and did not have surveillance.

## Methods

For this nationwide, retrospective cohort study, we assessed outcomes of current gynaecological surveillance in Dutch Lynch syndrome carriers: namely, the number of diagnosed endometrial carcinomas in cohorts with and without surveillance, their stage, and the advised additional adjuvant therapy in endometrioid endometrial carcinoma FIGO IA/IB, according to the current Dutch guideline.^24^ Index carriers with endometrial carcinoma before Lynch syndrome diagnosis were analyzed as a separate group.

### Patient selection

In case of a molecular Lynch syndrome diagnosis in The Netherlands, carriers are offered to register with The Netherlands Foundation for detection of Hereditary Tumors (*StOET*), starting in 1985. This foundation prospectively monitors to what extent registered carriers of an hereditary predisposition to cancer receive cancer-preventive care. For this purpose, the StOET sends reminders to clinicians involved in Lynch syndrome surveillance programs and keeps track of surveillance outcomes by regularly asking hospitals for these outcomes. In case the StOET does not have information about whether or not a carrier is enrolled in a gynaecological surveillance program, the gynaecology department of the hospital where this carrier underwent colonoscopies is asked for this information, as well as the general practitioner. If, upon multiple attempts, no response was obtained, we assumed patients received no surveillance. Additionally, the StOET tracks performance of (prophylactic) oncological surgeries and carriers’ date and cause of death. At the moment of data extraction, February 28th 2022, the StOET database contained 1908 germline proven Lynch syndrome carriers, who all granted informed consent to be included in the StOET database and to use their anonymized data for research purposes. Of these, 1255 were female. An overview of the StOET collaborative investigators can be found in Supplementary Table S1.

To ensure that we retrieved all pathological outcomes of registered Lynch syndrome carriers, data from all Lynch syndrome carriers was linked to data from the Dutch Nationwide Pathology Databank (PALGA). PALGA collects Dutch pathology reports from 1971 onwards and has a nationwide coverage since 1991. Reports of endometrial biopsies were used to supplement the StOET database.

As mentioned before, carriers with endometrial carcinoma prior to Lynch syndrome diagnosis were analyzed as a separate group, as it is unclear if the prognosis of index cases differs from those with Lynch syndrome -associated tumors after they have been diagnosed with Lynch syndrome.

### Data extraction

Based on the StOET database and PALGA information, we determined which eligible women had been enrolled in a gynaecological surveillance program over time. As we defined surveillance as an “ongoing evaluation of an individual with an increased risk of developing a disease”,^26^ women were classified as “having surveillance” if they had at least two gynaecological surveillance visits between 40 and 60 years of age, and had not had a hysterectomy at that time.^18^ If no information was available regarding surveillance visits for women during age 40–60, these women were considered not to be enrolled in a gynaecological surveillance program. If women opted out of surveillance after only one gynaecological visit or opted for prophylactic surgery after their first gynaecological visit, they were considered as “no surveillance”. Baseline characteristics for carriers were extracted from the StOET database, pathology-related characteristics were retrieved from the PALGA database. In patients with endometrial carcinoma FIGO IA and IB according to the 2008 FIGO guidelines, current guidelines recommend adjuvant brachytherapy and external radiotherapy, based on age of diagnosis, presence of LVSI, and endometrial carcinoma grade.^24^ Therefore, endometrial carcinomas were reclassified according to the FIGO 2017 stages,^27^ and for endometrioid FIGO stage IA and IB endometrial carcinomas requirement for adjuvant therapy was assessed.^24^

Additionally, the uptake of prophylactic gynaecological surgery was assessed. Hysterectomies with or without bilateral salpingo-oophorectomy were considered prophylactic in case corresponding carriers did not have a prior diagnosis of endometrial carcinoma.

### Ethics

Permission of the Erasmus Medical Center Committee on Research Involving Human Subjects was granted (MEC-2022-0809).

### Statistical analyses

Data were analyzed in SPSS statistical software, version 28.0. We described the number of observed endometrial carcinoma diagnoses in Lynch syndrome carriers. Differences in baseline characteristics were assessed by a χ^2^ test (Fisher's exact test) or Mann–Whitney U test, for categorical and quantitative variables, respectively. Carriers with unknown date of Lynch syndrome diagnosis, death, or endometrial carcinoma diagnosis, were excluded for further analyses. Kaplan–Meier time to event analyses were performed to assess survival from date of endometrial carcinoma diagnosis to either date of death or assembly of database (February 28th 2022), whichever came first. Survival estimates for five and ten year follow-up were calculated by log rank test and extracted from the Kaplan Meier curve. Due to the low number of subjects within each group, no attempt was made to adjust for covariates. Survival estimates were presented with the respective 95% confidence interval (95% CI). Cumulative incidences were calculated while accounting for the competing risks death and prophylactic hysterectomy. Graphs were plotted in R version 4.2.1. p-values <0.05 were considered statistically significant.

### Role of funding source

No funding source.

## Results

### Carriers

**Fig. 1:**
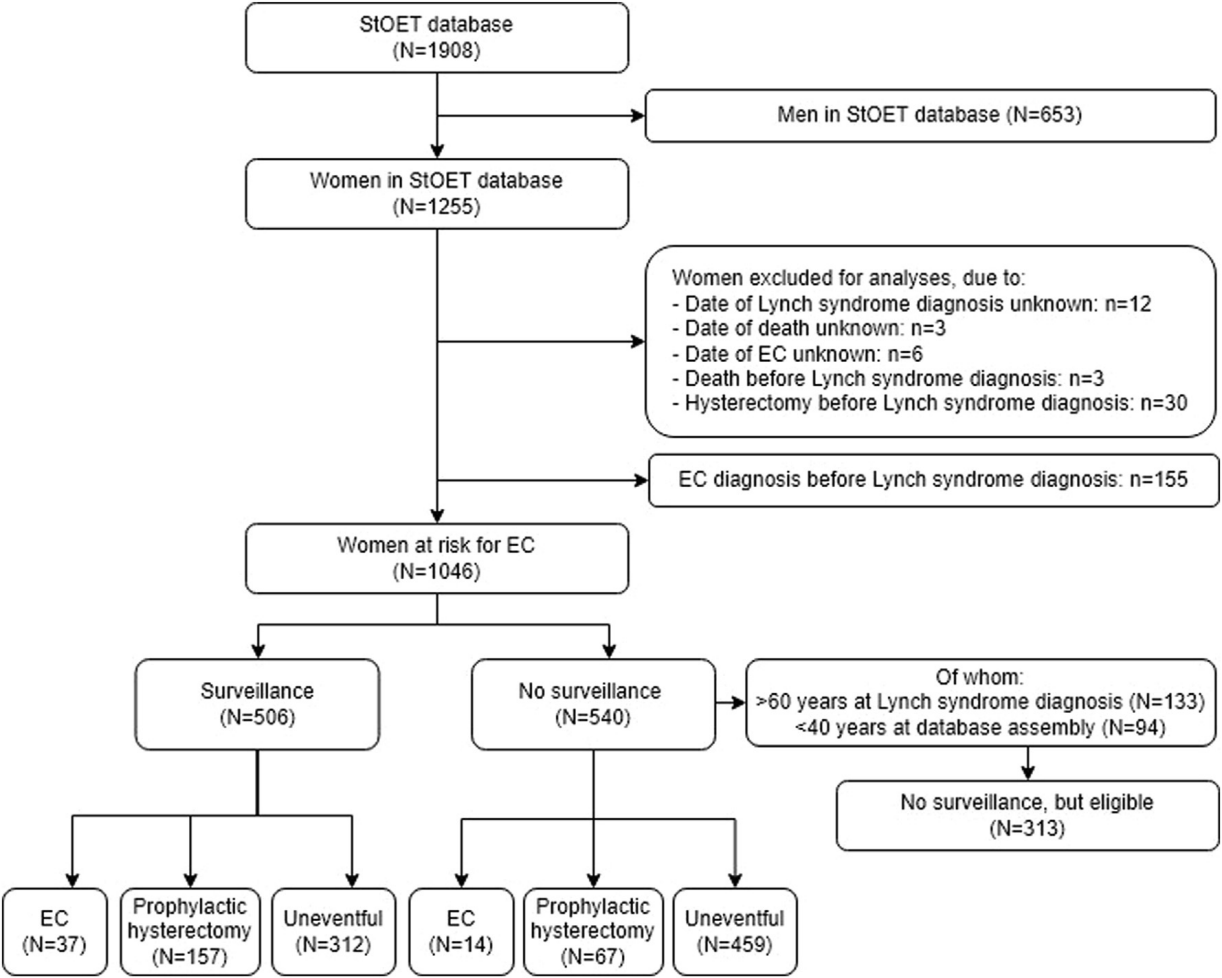
**Flowchart of Lynch syndrome carriers included in our study**.

In total, 1908 germline proven Lynch syndrome carriers were registered in the StOET database, of whom 1255 were women (Fig. 1). Of those, 209 women were not included in our “at risk for endometrial carcinoma group” due to various reasons: either lacking data, death or hysterectomy before Lynch syndrome diagnosis, or an endometrial carcinoma diagnosis prior to Lynch syndrome germline diagnosis (N = 155, Fig. 1). Of the remaining 1046 carriers at risk for endometrial carcinoma, 540 carriers did not have surveillance whatsoever, while 506 carriers did have surveillance. Carriers with surveillance were more often *MLH1* and *MSH2* carriers, while the no surveillance group contained relatively more *MSH6* carriers, although this difference was not statistically significant (154 *MLH1,* 164 *MSH2,* 145 *MSH6,* 42 *PMS2* carriers in surveillance group versus 142, 160, 169, 53 in no surveillance group, respectively, p = 0.32; Table 1). Uptake of surveillance was highest in *MLH1* carriers (154 out of 296 *MLH1* carriers, 52.0%) and lowest in *PMS2* carriers (42 out of 95 *PMS2* carriers, 44.2%). Median age at database assembly was 56 years (IQR 48–65 years) versus 65 years (IQR 49–75 years) in the surveillance versus no surveillance group, respectively (p < 0.0001). Carriers with surveillance were mostly diagnosed with Lynch syndrome under age 40 or between age 40 and 60, for carriers without surveillance this was mostly between age 40 and 60 (p < 0.0001).

**Table 1:** Baseline characteristics of total cohort of female Lynch carriers at risk for endometrial carcinoma (n = 1046).

	Surveillance (n = 506)	No surveillance (n = 540)	p-value
MMR gene involved			0.32
*MLH1*	154	142	
*MSH2*	164	160	
*MSH6*	145	169	
*PMS2*	42	53	
Unknown	1	16	
Age at lynch syndrome diagnosis, N			<0.0001
<40 years	244	180	
40–60 years	239	230	
>60 years	23	130	
Age at database assembly, median [IQR]	56 [48–65]	65 [49–75]	<0.0001
Years of follow up, median [IQR]	12 [6–18]	10 [4–16]	N/A
Women years of follow up, total	6151	5432	N/A
Women years in MLH1 carriers	2298	1783	
Women years in MSH2 carriers	2188	1672	
Women years in MSH6 carriers	1374	1653	
Women years in PMS2 carriers	291	324	
EC, total N (%^a^)	37 (7.3%)	14 (2.6%)	N/A
Hyperplasia (%^a^)	28 (5.5%)	4 (0.7%)	N/A
Age at EC diagnosis, median [IQR]	49 [46–55]	55 [49–67]	0.68
Years from DNA diagnosis until EC, median [IQR]	5 [2–9]	1 [0–4]	N/A
Prophylactic hysterectomy, N (%^a^)	157 (31.0%)	67 (12.4%)	<0.0001
Age at prophylactic hysterectomy, median years [IQR]	50 [43–56]	48.0 [42–57]	0.056
Age at prophylactic hysterectomy, N			0.288
Age <40 years, N	15	6	
Age 40–60 years, N	128	50	
Age >60 years, N	14	11	
Years from DNA diagnosis until prophylactic hysterectomy, median [IQR]	5 [2–10]	1 [0–5]	N/A
Death during follow-up, N (%^a^)	20 (4.0%)	94 (17.4%)	N/A
Years until death from DNA diagnosis, median [IQR]	10.5 [7.5–15.5]	6 [2–11]	N/A

a Percentages of total cohort with or without surveillance; EC, endometrial carcinoma; N/A, not applicable, as values were (indirectly) assessed in Kaplan Meier time-to-event analyses.

### Surveillance

Of the 1046 carriers at risk for endometrial carcinoma, 313 eligible carriers -those over age 60 at time of DNA diagnosis or under age 40 at time of database assembly excluded- did not have surveillance (30.0%; Fig. 1). In those carriers with surveillance, 37 endometrial carcinomas (out of 506 carriers, 7.3%) were diagnosed during surveillance, versus 14 endometrial carcinomas (out of 540 carriers, 2.6%) in those without (Table 1, Table 2; Supplementary Table S2). In both groups, the number of cases with hyperplasia was 28 (out of 506 carriers, 5.5%) and four (out of 540 carriers, 0.7%), respectively. In the surveillance group, only two endometrial carcinomas were diagnosed under age 40 and five above age 60, versus zero and six in the no surveillance group, respectively (Table 2).

**Table 2:** Characteristics of endometrial carcinomas found in females registered in the StOET database.

	Surveillance (n = 506)	No surveillance (n = 540)	EC before Lynch diagnosis (n = 155)
EC, N (%)	37 (7.3)	14 (2.6)	155
Age at EC diagnosis, N			
<40 years	2	0	19
40–60 years	30	8	117
>60 years	5	6	19
ECs based on MMR gene involved, N (% of known)			
*MLH1*	13 (35.1)	2 (14.3)	31 (20.1)
*MSH2*	18 (48.6)	5 (35.7)	49 (31.8)
*MSH6*	5 (13.5)	7 (50)	61 (39.6)
*PMS2*	1 (2.7)	0	13 (8.4)
Unknown	0	0	1
FIGO, N (% of known)			
IA	20 (62.5)	6 (46.2)	77 (57.5)
IB or higher	12 (37.5)	7 (53.8)	57 (42.5)
Unknown	5	2	21
Type of EC, N (% of known)			
Endometrioid EC	33 (94.3)	12 (85.7)	128 (88.3)
Non-endometrioid EC	2 (5.7)	2 (14.3)	17 (11.7)
Unknown	2	0	10
Adjuvant therapy for endometrioid EC FIGO IA/IB, N (% of known)			
None	21 (91.3)^a^	7 (87.5)^a^	63 (79.7)
Radiotherapy	1 (4.3)^a^	1 (12.5)^a^	10 (12.7)
Brachytherapy	1 (4.3)^a^	0^a^	2 (2.5)
Both	0^a^	0^a^	4 (5.1)
Unknown or not applicable	14	6	76

EC, endometrial carcinoma.

a No adjuvant therapy versus adjuvant therapy in no surveillance versus surveillance group, p = 1.

### Endometrial carcinomas

Endometrial carcinomas were found in FIGO stage IA in 62.5% (20 out of 32 with known grade) in carriers with surveillance versus in 46.2% (six out of 13 with known grade) of carriers without surveillance (Table 2). In both groups, endometrial carcinomas were predominantly of the endometrioid type (33 out of 35 with known histology, 94.3% and 12 out of 14, 85.7%). In carriers with surveillance, endometrial carcinomas were found at a significantly earlier age than in those without (median age at endometrial carcinoma diagnosis 49 years (IQR 46.0–55.0 years) versus 55 years (IQR 49.0–65.0 years); p = 0.68; Table 1). Adjuvant radiotherapy for endometrioid endometrial carcinomas FIGO stages IA and IB was only required twice (external beam and brachytherapy) in the surveillance group and once (external beam) in the no surveillance group (p = 1; Table 2).

### Endometrial carcinomas before Lynch syndrome diagnosis

As mentioned before, index carriers were analyzed as a separate group. Of the 155 carriers with endometrial carcinoma before Lynch syndrome diagnosis, 19 endometrial carcinomas were diagnosed before age 40 and 19 after age 60 (Table 2). Of note, approximately half of the 155 carriers (N = 76) still had had surveillance before endometrial carcinoma diagnosis (data not shown), possibly due to a positive family history. Endometrial carcinomas in this group were diagnosed in FIGO stage IA in 57.5% (77 out of 134 with known stage) and endometrial carcinomas were predominantly of endometrioid type (128 out of 145 with known histology, 88.3%; Table 2). However, more endometrioid endometrial carcinomas required adjuvant radiotherapy (16 out of 79 with data on adjuvant therapy, 20.3% in EC before Lynch syndrome diagnosis versus one out of eight, 12.5% in no surveillance group, versus two out of 23, 8.7% in surveillance group).

### Cumulative incidence of endometrial carcinomas

**Fig. 2:**
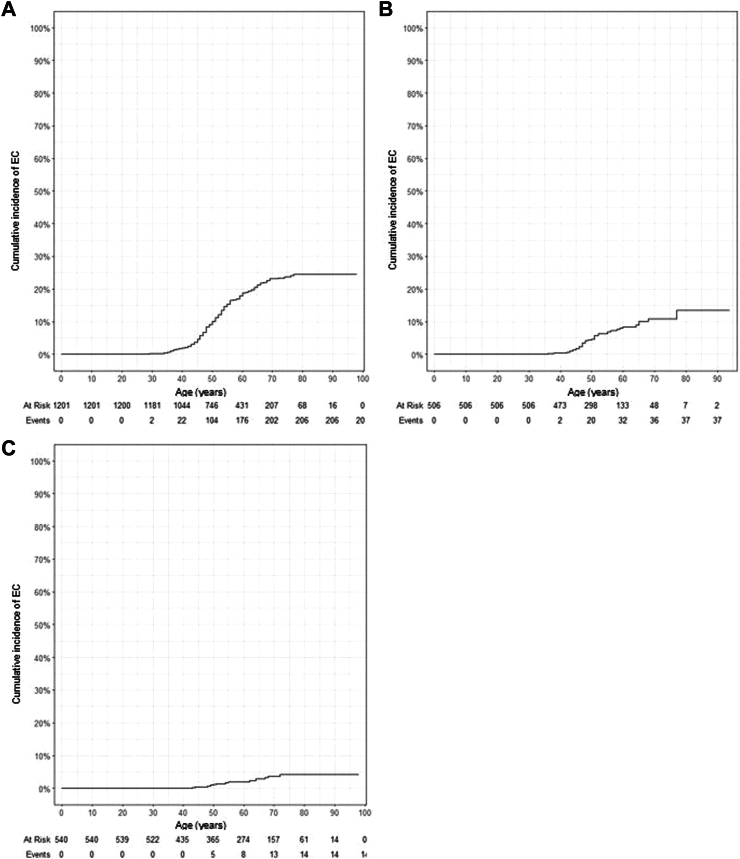
**Cumulative incidence for EC**. (A) All carriers, including index carriers. (B) Carriers with endometrial carcinoma after Lynch syndrome diagnosis with surveillance. (C) Carriers with EC after Lynch syndrome diagnosis without surveillance.

In all included female Lynch syndrome carriers together, regardless of surveillance and order of Lynch syndrome and endometrial carcinoma diagnosis, the cumulative incidence was 22.7% at age 70 (when accounted for cumulative risks death and prophylactic hysterectomy, Fig. 2A). When only cases with endometrial carcinoma after Lynch syndrome diagnosis were assessed according to surveillance status, cumulative incidence was 10.8% and 2.7% at age 70, for surveillance and no surveillance, respectively (Fig. 2B and C).

### Prophylactic hysterectomies

In carriers with surveillance, 31.0% (N = 157 out of 506) eventually opted for prophylactic hysterectomy, versus 12.4% (N = 67 out of 540) in the no surveillance group (Supplementary Table 3). When only eligible carriers -those >40 years of age at time of Lynch syndrome DNA diagnosis- were assessed, 224 (21.4% of all 1046 at risk) carriers chose for prophylactic hysterectomy (Table 1, Fig. 1). Most carriers chose for prophylactic hysterectomy when aged between 40 and 60 years (median in surveillance group 50 years (IQR 43.0–56.0 years) versus 48 years (IQR 42.0–57.0 years) in no surveillance group). When assessed per DNA MMR gene, uptake of prophylactic hysterectomy, regardless of surveillance, was highest in *MSH6* carriers (84 out of 314, 26.8%), followed by *PMS2* (25 out of 95, 26.3%) and *MLH1* carriers (56 out of 296, 18.9%), and lowest in *MSH2* carriers (58 out of 324, 17.9%). Of note, one OC was identified in a prophylactic surgery specimen in a woman without complaints.

### Survival

**Fig. 3:**
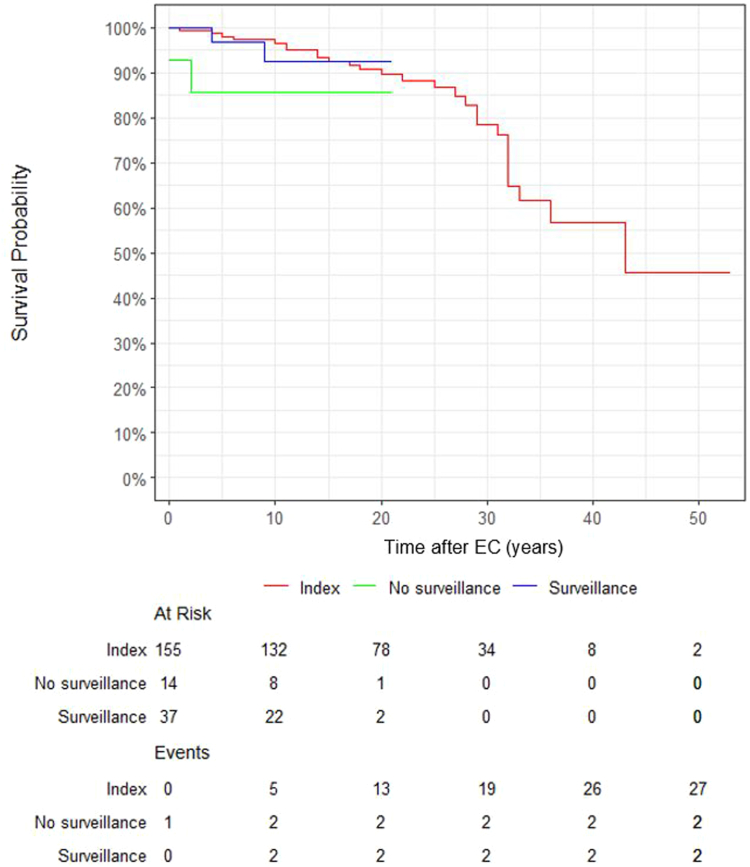
**Overall survival**. Overall survival in years of carriers with surveillance (blue line), without surveillance (green line), and carriers with endometrial carcinoma before Lynch syndrome diagnosis (purple line), from endometrial carcinoma onwards.

Of carriers with surveillance, 4.0% (20 out of 506) died during follow-up, compared to 17.4% (94 out of 540) of those without surveillance (Table 1). Only two carriers died due to endometrial carcinoma in each group; cause of death in both groups was predominantly related to other cancers (data not shown). Overall survival of carriers with endometrial carcinoma diagnosis with surveillance did not differ significantly from survival in carriers without, or survival of carriers with endometrial carcinoma before Lynch syndrome diagnosis (p = 0.51, Fig. 3). Overall survival five years after endometrial carcinoma diagnosis was 96.7% (95% CI 90.2%–100%) in carriers with surveillance, versus 86.7% (95% CI 69.5%–100%) in carriers without surveillance, versus 98.0% (95% CI 95.6%–100%) in carriers with endometrial carcinoma before Lynch syndrome diagnosis; ten-year survival was 92.5% (95% CI 82.3%–100%) in carriers with surveillance versus 96.4% (95% CI 93.6%–99.4%) in carriers with endometrial carcinoma before Lynch syndrome diagnosis. Of note, 76 carriers with endometrial carcinoma before Lynch syndrome diagnosis still had had surveillance, although surveillance in this group did not lead to significantly different survival (p = 0.43, data not shown).

## Discussion

In this retrospective cohort study, we described outcomes of gynaecological surveillance in a nationwide, prospectively maintained cohort of Dutch female Lynch syndrome carriers with and without gynaecological surveillance. In those carriers eligible for surveillance according to current guidelines, nearly one-third of carriers did not have surveillance. In surveillance, more endometrial carcinomas were diagnosed, on average more often in FIGO stage IA. However, overall survival did not differ significantly between carriers with and without surveillance and index carriers. Also, when only assessing FIGO stage IA and IB endometrioid endometrial carcinomas, surveillance did not seem to lead to substantially less carriers requiring adjuvant radiotherapy. Of all eligible carriers, nearly one-fourth eventually chose for prophylactic surgery, mostly between age 40 and 60 years. Cumulative incidence of all endometrial carcinomas, despite index status, was 22.7% at age 70.

Contradictorily, in carriers with surveillance, more endometrial carcinomas and hyperplasias were diagnosed. This phenomenon was recently also described in CRC.^28^ Here, authors suggest that one of the mechanisms for this could be over diagnosis of CRCs that would have otherwise not been diagnosed, for example due to attacking of microsatellite unstable crypts by a person's own immune system.^29^ Currently, little is known about the carcinogenesis in Lynch syndrome-associated endometrial carcinoma, but a study by Niskakoski et al.^30^ showed that MMR deficiency could be found even in early lesions preceding endometrial carcinoma. An over diagnosis of hyperplasias and early endometrial carcinomas, which would have otherwise been eradicated by the body's immune system without any interference, could explain the higher proportion of lesions in our surveillance group as well. Additionally, this phenomenon could be explained by lead-time or detection bias: surveillance causes earlier detection of endometrial carcinomas, that would have been diagnosed later in life. It is also possible that the growth of some (indolent) tumors is too slow to cause complaints before carriers die due to other causes (which was accounted for in the endometrial carcinoma cumulative incidence, but not in the absolute number of endometrial carcinomas). Besides this, the more endometrial carcinomas and hyperplasias in the surveillance group could also at least partially be explained by the differences in baseline characteristics between the surveillance and no surveillance group in our study. The group without surveillance was on average significantly older at database assembly and at Lynch syndrome diagnosis than the group with surveillance, which could also be explained by the fact that this group contained more carriers aged >60 years at the time of DNA diagnosis, and these women were therefore not eligible for surveillance anymore (confounding by indication). Similarly, women enrolled in our study without a prior endometrial carcinoma at age 60 have a lower chance to still develop an endometrial carcinoma than at age 25 (immortal time bias). Second, the group with surveillance contained relatively more *MLH1*/*MSH2*/*EpCAM* carriers, albeit not significant, who have a higher risk to develop endometrial carcinoma,^31^ usually have an earlier endometrial carcinoma age of onset,^32^ and were historically advised to enroll in a gynaecological surveillance program from the age of 30–35 years old (no time-trend bias). Also, family history -also partially corresponding with MMR gene involved- could have affected carriers to choose for surveillance or not. However, a recent study by Sun et al,^33^ found that family history negatively correlated to the extent of carriers choosing for surveillance, albeit groups were small. Nevertheless, unfortunately, this was not assessed in our study. As a combination of these factors probably affected constitution of both groups, results of this study should be interpreted carefully.

Strikingly, nearly one-third of eligible carriers did not have (ongoing) surveillance. Due to the retrospective nature of this study, it is possible that part of these carriers were enrolled in a gynaecological surveillance program, but were not registered as being so. However, the StOET is a prospectively maintained database, where clinicians taking care for Lynch syndrome carriers (amongst whom gynaecologists) are asked each year if carriers have already had their surveillance visit, and what these visits' outcomes were. We therefore deem chances small that carriers registered as having no surveillance were or are actually enrolled in a gynaecological surveillance program. Future research should focus on why these women do not have gynaecological surveillance; and to what extent this is based on a personal choice or not being offered gynaecological surveillance, for example. Previous research showed that endometrial biopsies are considered painful and this was the main reason to quit surveillance as may have been the case in the carriers in our cohort, who only had one gynaecological visit.^34^ However, this does not apply to the women without surveillance whatsoever, although one can imagine that a negative experience of family members might affect one's choice to opt for surveillance. Another cause for not choosing surveillance, could be found in a negative family history for endometrial carcinoma or the fact that generally, endometrial carcinomas present with early irregular or postmenopausal blood loss.^35^ A small study published in 2017 found that the majority of endometrial carcinomas were diagnosed due to gynaecological complaints, instead of due to surveillance.^36^ Also, although all carriers have been counseled by a clinical geneticist, aware of the endometrial carcinoma risks, content of the counseling might vary. Some counselors might not advocate for gynaecological surveillance, as its effect is not proven. Furthermore, carriers might have forgotten about this information as they had been diagnosed with Lynch syndrome years earlier (more than 40%, 424 out of the 1046 carriers, received a Lynch syndrome diagnosis before age 40). After a Lynch syndrome diagnosis, patients are advised to visit a gynaecologist, but they might not actively be recalled by the hospital. Finally, different death rates in surveillance and non-surveillance group might explain in part why carriers opted out for surveillance, as they might had already been diagnosed with another (high-stage) Lynch syndrome -associated tumor, such as CRC.

Endometrial carcinoma diagnosis seemed to be slightly earlier (relatively more FIGO stage IA) in carriers with compared to those without surveillance, but did not lead to substantially more adjuvant therapy. Current guidelines advise adjuvant therapy in case of median and high risk low stage endometrioid endometrial carcinoma, and specifically external radiotherapy is known to cause significant morbity.^37^ Interestingly, recent studies showed a limited benefit of radiotherapy in stage I mismatch repair deficient (MMRd) endometrioid endometrial carcinoma and a significantly worse overall survival in early stage MMRd endometrioid endometrial carcinomas treated with adjuvant brachytherapy alone.^38^^,^^39^ Besides a decrease in mortality and number of endometrial carcinoma diagnoses (prevention by prophylactic hysterectomy in case of for example hyperplasia), surveillance aims to decrease the number of carriers requiring this type of adjuvant therapy. Also, some gynaecologists argue surveillance should be performed to not only detect endometrial carcinoma, but to predominantly detect hyperplasia. In this study, also more cases with hyperplasia were found in the group with surveillance; by performing a hysterectomy in these carriers, outgrowth of endometrial carcinoma can still be prevented.

In total, cumulative incidence of all endometrial carcinomas, regardless of index status, was 22.7%. This was lower than previously reported.^8^^,^^31^^,^^40^^,^^41^ This could possibly be explained by ascertainment bias: previously, specifically those carriers with high tumor risks and/or highly positive family history were assessed, whereas nowadays (also with routine immunohistochemistry screening in all endometrial carcinomas under age 70) also Lynch syndrome carriers with a lower risk of Lynch syndrome-associated tumors are diagnosed. Therefore, it is possible that Lynch syndrome-associated tumor risks used to be higher, and gradually decreased while also lower-risk Lynch syndrome carriers are assessed. Second, previous studies reported on endometrial carcinomas diagnosed in multiple countries, with divergent surveillance recommendations (with regards to both interval as methods).^12^^,^^20^^,^^21^^,^^41^ As mentioned above, in The Netherlands, in case of hyperplasia, endometrial carcinoma outgrowth is prevented as a prophylactic hysterectomy is advised.

In 21.4% (224 of all 1046 included carriers), carriers opted for prophylactic hysterectomy, mostly between age 40 and 60 years, as was expected. Most carriers underwent surveillance first for approximately five years and then chose for risk-reducing surgery. This phenomenon could be explained by the fact that endometrial biopsies are considered painful and a reason to quit surveillance, as mentioned previously.^34^ Secondly, women who visit a gynaecologist are better informed about risk-reducing surgery and might be aware that due to the general acceptance of laparoscopic procedures, the burden and morbidity of such surgery is acceptable. Also, it may be harder to decide on the specific type of risk-reducing surgery (with/without salpingo-oophorectomy) for women aged just above 40; in contrast, for those closer to the age of biological menopause, this decision seems easier. Obviously, the fact that more carriers with surveillance chose for risk reducing surgery could be biased by the fact that both groups differed in baseline characteristics, although a recent study showed that there is no correlation between endometrial carcinoma gene and age risk estimates and the choice for risk reducing surgery.^42^ This was also found in our study, as the highest uptake of prophylactic surgery, regardless of enrollment in surveillance, was found for *MSH6* carriers, followed by *PMS2* and *MLH1* carriers, with lowest uptake in *MSH2* carriers. This could be partially explained by the fact that initially, *MSH6* carriers were thought to have a higher endometrial carcinoma risk and were thus more actively counselled to have a prophylactic hysterectomy in The Netherlands. Remarkably, the second-most prophylactic surgeries were performed in *PMS2* carriers. The reason for this remains unclear, as Dutch clinical geneticists and gynaecologists are in practice more hesitant to advice these carriers prophylactic surgery due to their relatively lower cancer risks.

Of note, in only one hysterectomy specimen, endometrial carcinoma was identified, suggesting that prophylactic surgery was performed in time in the majority of carriers. Similarly, one OC was diagnosed by chance in prophylactic surgery tissue.

Survival after endometrial carcinoma diagnosis was also assessed in carriers with surveillance, those without, and those with endometrial carcinoma before Lynch syndrome diagnosis. Overall survival did not differ significantly between these three groups. Of note, we adjusted for competing risks death and prophylactic hysterectomy. Also, it is important to keep in mind that immortality bias plays a role here, as carriers with endometrial carcinoma preceding Lynch syndrome diagnosis would have been excluded from this analysis in case of prior death. Although overall survival after endometrial carcinoma diagnosis did not seem to differ between carriers with and without surveillance, both groups were relatively small with limited events (death or hysterectomy). Therefore, these results might not reflect true survival in a larger group of Lynch syndrome carriers post-endometrial carcinoma diagnosis, and should be interpreted with care. We do know, however, that overall survival after endometrial carcinoma is generally relatively good, specifically for FIGO IA endometrial carcinomas (5-year OS >90%),^43^ which represent more than half of endometrial carcinomas found in women after Lynch syndrome diagnosis, regardless of enrollment in gynaecological surveillance programs or not. More research should therefore be performed to assess to what extent this survival difference remains non-significant, also taking into account other Lynch syndrome -associated cancers.

Similarly, as approximately half of the index carriers with endometrial carcinoma still had undergone surveillance, survival did not differ significantly between those index carriers with and those without surveillance. Since detection of endometrial cancer and the overall survival of endometrial cancer itself is relatively good, only very large datasets will be able to show benefit of early detection of endometrial carcinoma or hyperplasia on either outcome. Although this is a relatively large cohort, these numbers are not met. Only collaboration between different countries will be able to truly answer these questions, as for this survival analysis, groups were too small to draw firm conclusions. Assessing survival in these carriers is challenging, as Lynch syndrome carriers with endometrial carcinoma preceding Lynch syndrome diagnosis could have another cancer risk. Similarly, these carriers cannot be classified as having had no gynaecological surveillance, as half of them still had some form of gynaecological surveillance after all, probably based on a positive family history for endometrial carcinoma (without a molecular Lynch syndrome diagnosis yet). Therefore, we chose to depict survival of index cases apart from survival of carriers with endometrial carcinoma and surveillance and survival of carriers with endometrial carcinoma without surveillance.

As it is currently not clear to what extent gynaecological surveillance for female Lynch syndrome carriers contributes to a better survival,^17^ alternatives should be assessed. Although a recent modeling study^44^ showed that gynaecological surveillance could be cost-effective, larger studies should be performed to assess this in practice. Here, also morbidity of surveillance should be assessed. Meanwhile, (less invasive) alternatives for the current form of surveillance should be explored. Female Lynch syndrome carriers should be counseled extensively, discussing the limited evidence for current forms of gynaecological surveillance and personal factors such as their endometrial carcinoma risk (based on MMR gene involved), age, and expectations of surveillance and preventative surgery. Similarly, alternatives for current gynaecological surveillance should be assessed: for example, as mentioned by Ryan et al,^17^ the eligibility and safety of regular tele-health appointments, where the presence of occult blood loss and other endometrial carcinoma-accompanying complaints will be discussed. When developing new gynaecological surveillance alternatives, female carriers should be asked what kind of surveillance they would prefer and reasons why women previously choose to opt out for surveillance (such as for example discomfort and inconvenience) should be considered.

Our study had several limitations. First, we were hindered by the number of Lynch syndrome carriers registered in the StOET database. Therefore, this study was not powered and outcomes of this study are mainly descriptive. Second, despite being prospectively maintained, part of the data are retrospectively analyzed, which might have introduced bias regarding carriers being enrolled in a surveillance program or not. Similarly, data comprise a large timespan in which surveillance guidelines were revised and surveillance techniques changed. Third, we expect that multiple confounding factors affected our study outcomes, as both groups were not similar in composition. Fourth, our study outcomes might not be generalizable to all countries, as the care for Lynch syndrome carriers is organized differently in each country, with some countries not actively advising gynaecological surveillance. Therefore, our results should be interpreted with care. Regardless, to our knowledge, this is the first study to compare outcomes of gynaecological surveillance in a large cohort of molecularly diagnosed female Lynch syndrome carriers with and without surveillance.

In conclusion, we found that nearly one-third of Lynch syndrome carriers in a nationwide cohort did not have gynaecological surveillance while eligible. Endometrial carcinomas seemed to be diagnosed in a slightly earlier stage during surveillance, although this did not seem to substantially decrease the requisite for adjuvant therapy or affect overall survival, questioning the effectiveness of current Dutch gynaecological surveillance with regards to long-term survival for female Lynch syndrome carriers. Future prospective studies are warranted to conclusively determine to what extent gynaecological surveillance in its current form contributes to earlier detection and possibly even lower mortality and morbidity of Lynch syndrome-associated endometrial carcinoma. Additionally, alternatives for current gynaecological surveillance should be assessed, which should also consider patient preferences.

## Contributors

Conceptualization: HCvD, AW; Data curation: ELE, LvL; formal analysis: ELE, HCvD, AW; funding acquisition: AW, MCWS; investigation: ELE, LvL; methodology: HCvD, AW; project administration: AW; resources: FG, MEvL, AMvA, JMW; supervision: MCWS, HCvD, AW; validation: MEvL, HCvD, AW; visualisation: HCvD, AW; writing—original draft: ELE, AW; and writing—review & editing: ELE, LvL, FG, JMW, AMvA, MEvL, MCWS, HCvD, AW.

All authors read and approved the final version of the manuscript. ELE, LvL, HCvD, and AW accessed and verified the data.

## Data sharing statement

Data from this study are not publicly available. De-identified participant data used in this study will be shared (via SURFfilesender) with researchers who submit a research proposal, upon approval of the proposal by the Erasmus MC and StOET Research Board, with publication. Additional related documents will not be made available.

## Declaration of interests

MCWS received research support from Sysmex, Sentinel, and Norgine. MEvL received an honorarium for guest editor and writing a review *Best Practice Research* Clinical Gastroenterology in 2022. The remaining authors declare no competing interests.
