# Supplementary material for: Outcomes of endometrial cancer prevention strategies in patients with Lynch syndrome: a nationwide cohort study in the Netherlands
Source: eClinicalMedicine. 2024 Dec 21;79:103006. doi: 10.1016/j.eclinm.2024.103006 (PMC11733057; doi:10.1016/j.eclinm.2024.103006)
Supplement: Supplementary material [file mmc1.docx]

**Supplementary Table 1** Consortium StOET Study Group

| M.C. |  | Breijer |
| --- | --- | --- |
| A.S. |  | Tjalsma |
| F. |  | Vork |
| H.P.M. |  | Smedts |
| J. | van der | Velden |
| M.M.A. |  | Brood-van Zanten |
| J.E. | van de | Riet |
| A.L.M. |  | Oei |
| H. |  | Kessel |
| P.M.L.H. |  | Vencken |
| M.P.L.M. |  | Snijders |
| R.H.M. |  | Hermans |
| A. |  | Bouman |
| H.W. |  | Ünsalan |
| A.M.G. | van de | Swaluw |
| G.M. |  | Plaisier |
| H.C. | van | Doorn |
| K. | van den | Berg |
| W. |  | Hofhuis |
| Y.A.J.M. |  | Dabekausen |
| P.R. |  | Kolk |
| H.T.C. |  | Nagel |
| A.M.L.D. | van | Haaften-de Jong |
| A.C. | van | Hof |
| M. | van den | Hende |
| J. |  | Kaijser |
| H.H. | de | Haan |
| R.A. |  | Smit |
| M.W.G. |  | Moonen-Delarue |
| J.J. |  | Beltman |
| J.E. |  | Martens |
| R. |  | Kruitwagen |
| J.M. | van der | Ploeg |
| J.M. |  | Woolderink |
| S.F.P.J. |  | Coppus |
| M.J. |  | Duk |
| M.J.A. |  | Apperloo |
| C.M. |  | Koopmans |
| C.C.M. |  | Buis |
| H. | van | Meurs |
| E.C. |  | Dul |
| B.B.J. |  | Hermsen |
| A.M. | van | Altena |
| A. |  | Baalbergen |
| A.A. | van | Ginkel-Terng |
| M. |  | Baas |
| P. | van | Greunen |
| C.M.W.H. | | Smeets |
| H. |  | Knipscheer |
| J.E. |  | Martens |
| C. |  | Schmeink |
| M.D. | van der | Laan |
| E.J.M. | van | Es |
| J.E.W. | van | Dijk |
| F.M.F. |  | Rosier-van Dunné |
| H. |  | Nijman |
| C.G. |  | Gerestein |
| D. |  | Boskamp |
| E.C.A.H. |  | Scheers |
| M. |  | Verbruggen |
| L.R. |  | Bartelink |
| C.B.M. |  | Kruijdenberg |
| J.M. |  | Briët |
| B. |  | Visschers |
| M. |  | Engelen |

**Supplementary Table 2** Characteristics of female Lynch syndrome carriers with endometrial carcinoma after Lynch syndrome diagnosis (n=51).

| MMR gene | Age at DNA diagnosis, years | Surveillance | Age at EC diagnosis, years | EC type | EC FIGO stage |
| --- | --- | --- | --- | --- | --- |
| *MLH1* | 37 | Yes | 46 | Endometrioid | 1A |
| *MLH1* | 52 | Yes | 58 | Non endometrioid | 1B |
| *MLH1* | 34 | Yes | 48 | Endometrioid | Unknown |
| *MSH2/EpCAM* | 57 | Yes | 77 | Endometrioid | 1B |
| *MSH2/EpCAM* | 38 | Yes | 45 | Endometrioid | Unknown |
| *MSH2/EpCAM* | 25 | Yes | 38 | Endometrioid | Unknown |
| *MSH2/EpCAM* | 38 | Yes | 43 | Endometrioid | 1B |
| *MLH1* | 45 | Yes | 55 | Endometrioid | 1B |
| *MSH2/EpCAM* | 63 | Yes | 65 | Endometrioid | 1B |
| *MLH1* | 47 | Yes | 64 | Non endometrioid | 1A |
| *MSH2/EpCAM* | 59 | No | 64 | Endometrioid | 2 |
| *MSH2/EpCAM* | 41 | Yes | 43 | Endometrioid | 1A |
| *MSH2/EpCAM* | 56 | No | 62 | Endometrioid | 2 |
| *MSH2/EpCAM* | 39 | Yes | 47 | Endometrioid | 1A |
| *MLH1* | 40 | Yes | 48 | Endometrioid | 1A |
| *MLH1* | 43 | Yes | 48 | Endometrioid | 1A |
| *MLH1* | 31 | Yes | 52 | Endometrioid | 1A |
| *MLH1* | 40 | Yes | 46 | Endometrioid | 1A |
| *MSH2/EpCAM* | 48 | No | 50 | Endometrioid | 1A |
| *MSH2/EpCAM* | 33 | Yes | 51 | Endometrioid | Unknown |
| *MSH2/EpCAM* | 47 | Yes | 49 | Endometrioid | 3C |
| *MSH2/EpCAM* | 43 | Yes | 44 | Endometrioid | 1A |
| *MSH2/EpCAM* | 40 | No | 43 | Endometrioid | 1B |
| *MSH6* | 68 | No | 68 | Non endometrioid | 4A |
| *MSH2/EpCAM* | 33 | Yes | 36 | Endometrioid | 1A |
| *MSH6* | 52 | No | 54 | Endometrioid | 1B |
| *MSH2/EpCAM* | 39 | No | 44 | Non endometrioid | 4B |
| *MSH2/EpCAM* | 44 | Yes | 50 | Endometrioid | 1A |
| *MSH6* | 45 | No | 49 | Endometrioid | 1A |
| *MSH6* | 50 | No | 51 | Endometrioid | 4B |
| *MSH2/EpCAM* | 41 | Yes | 44 | Endometrioid | 1A |
| *MSH6* | 47 | No | 48 | Endometrioid | 1A |
| *MSH2/EpCAM* | 52 | Yes | 56 | Endometrioid | 1A |
| *MSH2/EpCAM* | 50 | Yes | 51 | Endometrioid | 3C |
| *MLH1* | 54 | Yes | 68 | Endometrioid | 1A |
| *MLH1* | 52 | No | 55 | Endometrioid | 1A |
| *MSH6* | 59 | Yes | 59 | Endometrioid | 1A |
| *MSH6* | 59 | Yes | 60 | Endometrioid | 1A |
| *MLH1* | 43 | Yes | 47 | Endometrioid | 3C |
| *MSH6* | 50 | Yes | 51 | Unknown | 3C |
| *MSH2/EpCAM* | 51 | Yes | 52 | Endometrioid | 3A |
| *MSH6* | 44 | Yes | 51 | Endometrioid | 1A |
| *MLH1* | 63 | No | 72 | Endometrioid | Unknown |
| *MLH1* | 43 | Yes | 46 | Endometrioid | 1B |
| *MSH2/EpCAM* | 42 | Yes | 47 | Endometrioid | 2 |
| *MSH2/EpCAM* | 36 | Yes | 42 | Endometrioid | 1A |
| *MSH6* | 64 | Yes | 65 | Endometrioid | 1A |
| *MSH6* | 66 | No | 67 | Endometrioid | 1A |
| *PMS2* | 25 | Yes | 47 | Unknown | Unknown |
| *MLH1* | 49 | Yes | 55 | Endometrioid | 1A |
| *MSH6* | 64 | No | 64 | Endometrioid | 1A |

**Supplementary Table 3** Characteristics of the 67 women without surveillance, but with prophylactic hysterectomy.

| MMR gene involved  *MLH1*  *MSH2*  *MSH6*  *PMS2*  Unknown | 16  17  26  8  0 |
| --- | --- |
| Age at Lynch syndrome diagnosis, N  <40 years  40-60 years  >60 years | 14  44  9 |
| Age at database assembly, median [IQR] | 63 [53-68] |
|  |  |
| Years from DNA diagnosis until prophylactic hysterectomy,  median [IQR] | 1 [0-4] |
| Number of carriers with prophylactic hysterectomy <1 year, N (%) | 23 (34.3%) |
| EC at prophylactic hysterectomy, total N | 0 |
| Hyperplasia at prophylactic hysterectomy, N (%) | 3 (4.5%) |
|  |  |
|  |  |
| Death during follow-up, N (%*) | 3 (4.5%) |
| Years until death from DNA diagnosis, median | 14 |

^*^ % op 67 women.
